# Thrombophlebitis migrans in a man with pancreatic adenocarcinoma: a case report

**DOI:** 10.1186/1757-1626-2-6610

**Published:** 2009-04-29

**Authors:** Sreedhari Thayalasekaran, Helen Liddicoat, Eleanor Wood

**Affiliations:** 1 Broomfield Hospital, Court Road, Broomfield, Chelmsford CM1 7ET; 2 Deparment of Respiratory Medicine, Chase Farm Hospital, The Ridgeway, Enfield, Middlesex, EN2 8JL; 3 Department of Gastroenterology, Homerton University Hospital NHS Foundation Trust, Homerton Row, London, E9 6SR

## Abstract

**Introduction:**

Thrombophlebitis migrans is characterised by the development of recurrent (i.e. migratory) superficial thrombophlebitis. It is an acquired coagulopathy that is strongly associated with malignancy, especially solid tumours of the adenocarcinoma type.

**Case presentation:**

A 62 year old male presented with jaundice, abdominal pain, anorexia, steatorrhoea and dark urine. Ultrasound demonstrated a mass in the head of the pancreas causing common bile duct obstruction. Histology confirmed pancreatic adenocarcinoma. He was subsequently noted to have a migratory, tender and erythematous rash consistent with thrombophlebitis migrans.

**Conclusion:**

Thrombophlebitis migrans is more easily recognised in patients with an established diagnosis of malignancy than in situations where the thrombophlebitis is first diagnosed. In the latter situation, investigations for an occult malignancy should be sought.

## Introduction

Thrombophlebitis migrans is an acquired coagulopathy characterised by the development of recurrent (i.e. migratory) superficial thrombophlebitis. Approximately 50% of cases are linked to an underlying malignancy [1].

The strongest association is with solid tumours of the adenocarcinoma type. Pancreatic cancer, especially of the body or tail of the pancreas, seems to be associated with the highest risk of thrombophlebitis migrans [2].

The connection between malignancy and thrombophlebitis migrans was first described in 1865 by Professor Armand Trousseau (Trousseau's syndrome), in the diagnosis of his own pancreatic cancer [3].

## Case presentation

A 62-year-old Caucasian male presented with post-prandial abdominal pain, anorexia, jaundice, steatorrhoea and dark urine.

There was no recent history of foreign travel or unprotected sexual intercourse. There was no family history of malignancy reported. Other than an annual review for a treated thyroid toxic adenoma there was no significant past medical history. He was a smoker and drank alcohol in moderation. Prior to retirement he worked as a gardener. On physical examination he was apyrexial with jaundice of his skin and sclera. The abdomen was soft and non-tender, with associated hepatomegaly.

Imaging with ultrasound demonstrated a mass in the head of the pancreas causing common bile duct obstruction. Computerised Tomography (CT) showed multiple metastatic lesions within the liver. Endoscopic retrograde cholangio-pancreatography (ERCP) revealed ulceration in the second and third parts of the duodenum, suggestive of malignant infiltration. Histology confirmed pancreatic adenocarcinoma. To decompress the biliary tree and thus relieve the jaundice a stent was inserted into the common bile duct. The diagnosis was explained to the patient and he was discharged home. He was scheduled to see an oncologist and receive palliative chemotherapy as an outpatient.

A tender erythematous rash on the medial aspect of the right forearm and left lower limb was noted two months later during a planned admission for a blood transfusion. Doppler ultrasound of the arm did not reveal any evidence of deep vein thrombosis. The patient described rashes of a similar appearance in multiple areas over the preceding weeks. These findings were consistent with thrombophlebitis migrans. This was treated medically with non-steroidal anti-inflammatory drugs and intravenous antibiotics. During his course of chemotherapy, he developed neutropenic sepsis and passed away.

## Discussion

The typical lesions of thrombophlebitis migrans are recurrent tender erythematous nodules occurring in successive crops in the subcutaneous fat of the trunk or extremities [4].

It is believed that Trousseau's syndrome is due to chronic subclinical disseminated intravascular coagulation caused by the release of procoagulants from tumour cells involving tissue factor expression, as well as direct activation of platelets [5,6].

Other predisposing factors to the prothrombotic state include: bed rest, infection, surgery and anti-cancer drugs. This thromboembolic phenomenon is unresponsive to warfarin but can be managed with heparin-based anticoagulation [4].

A variety of conditions can present themselves with a similar appearance to Trousseau's syndrome. Differential diagnoses to consider are other conditions known to trigger hypercoagulable states:

**Figure 1:**
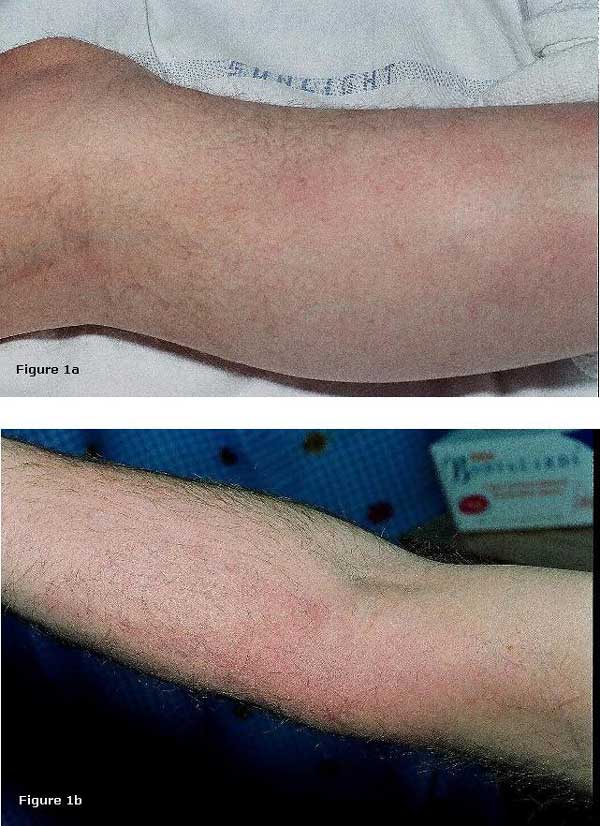
**(A) and (B) Tender erythematous rash on the medial aspect of the right forearm and left lower limb**.

Erythema nodosum, Lymphangitis, Vasculitis, Buerger's disease, and Behcet's disease [6].

A diagnosis of Trousseau's syndrome is far easier to make in a person with an established malignancy than when the thrombophlebitis is identified first. In the latter situation detection of the cancer is more difficult and consequently often delayed. In these situations, patients should undergo a thorough investigation for an occult malignancy, including upper endoscopy and CT of the chest, abdomen and pelvis, to exclude an underlying malignancy [6].

An additional sign of underlying malignancy in patients with idiopathic/unprovoked thromboembolism is resistance to anticoagulation with warfarin but response to heparin [4].

## Conclusion

It was easier to recognise thrombophlebitis migrans in this gentleman because the diagnosis of pancreatic adenocarcinoma was already established. Any patient that displays the characteristic migratory, tender and erythematous rash without an underlying diagnosis should be fully investigated to exclude malignancy.

## List of abbreviations

CT: Computer Tomography; ERCP: Endoscopic retrograde cholangio-pancreatography.

## Consent

Written and informed consent were obtained from the patient for publication of the accompanying images. A copy of the written consent is available for review by the Editor-in-Chief of this journal.

## Competing interests

The authors declare that they have no competing interests.

## Authors' contribution

ST was the principal author of the manuscript. HL was responsible for the preliminary draft. EW was responsible for the final proof reading of the case report.

## References

[B1] Lee AY Levine MN Venous thromboembolism and cancer: risks and outcomes Circulation 2003 107 117 121 10.1161/01.CIR.0000078466.72504.AC 12814981

[B2] Ogren M Bergqvist D Wahlander K Trousseau's syndrome-what is the evidence? A population-based autopsy study Thromb Haemost 2006 95 541 545 16525584 10.1160/TH05-10-0694

[B3] Lesher JL Jr Thrombophlebitis and thromboembolic problems in malignancy Clinical Dermatology 1993 11 159 163 10.1016/0738-081X(93)90113-Q 8339192

[B4] Levine M Treatment of thrombotic disorders in cancer patients Haemostasis 1997 27 1 38 43 9439757 10.1159/000217481

[B5] Wahrenbrock M Borsig L Le D Selectin-mucin interactions as a probable molecular explanation for the association of Trousseau syndrome with mucinous adenocarcinomas J Clin Invest 2003 112 853 862 10.1172/JCI18882 12975470 PMC193671

[B6] Resident Staff Issues articles 2007 http://www.residentstaff.com/issues/articles/2007-06 05.asp

